# Cyclothymia, bipolar disorder and multiple sclerosis: A case report

**DOI:** 10.1192/j.eurpsy.2023.1471

**Published:** 2023-07-19

**Authors:** M. D. P. Paz Otero, E. Lozano Bori, J. Sánchez Rodríguez, S. Puyal González, M. Fernández Fariña, F. Mayor Sanabria, A. Francos Ajona

**Affiliations:** 1 Psychiatric Acute Inpatient Unit; 2 Mental Health Center (Centro), Hospital Clínico San Carlos, Madrid, España, Madrid, Spain

## Abstract

**Introduction:**

We present the case of a 49-year-old woman who was diagnosed with multiple sclerosis at the age of 19 and suffers from an affective disorder that has been evolving for years. This condition, for which she has been followed by psychiatry and psychology for more than ten years, consists of alternating periods of hypomania lasting weeks and phases in which frank depressive symptomatology predominates, with no phases of euthymia in between and with a predominance of severe deterioration of her functionality at both poles.

**Objectives:**

(1) We will review the term cyclothymia and explore the concept of “cyclothymic temperament” advocated by some authors, in order to be able to understand the dimension of the present case and reformulate its approach.

(2) The relationship between multiple sclerosis and bipolar spectrum disorders will be covered, reviewing the current knowledge in this regard and relating it to the patient’s symptomatology.

**Methods:**

A review of the patient’s clinical history will be carried out, taking into account her life history, the complementary tests performed as well as the multiple therapeutic approaches tried over the last few years.

Likewise, a bibliographic review of the available scientific literature will be carried out in relation to the diagnosis of cyclothymia or bipolar disorder type II, the controversial term “cyclothymic temperament”, and the relationship that these diagnoses have with the diagnosis of Multiple Sclerosis.

**Results:**

(1) Our patient could fit into what many authors define as a cyclothymic temperament, fulfilling, in certain episodes, the criteria that the manuals propose for bipolar disorder type II.

**(2) 2.1** The prevalence of bipolar affective disorder in MS is approximately twice as high as in the general population (rates of 0.3-2.4%). **2.2** Patients with MS have higher scores in cyclothymic and hyperthymic temperament than the control group. **2.3** Certain drugs generally used in BD also seem to have a beneficial effect on MS.

**Image:**

**Figure:**
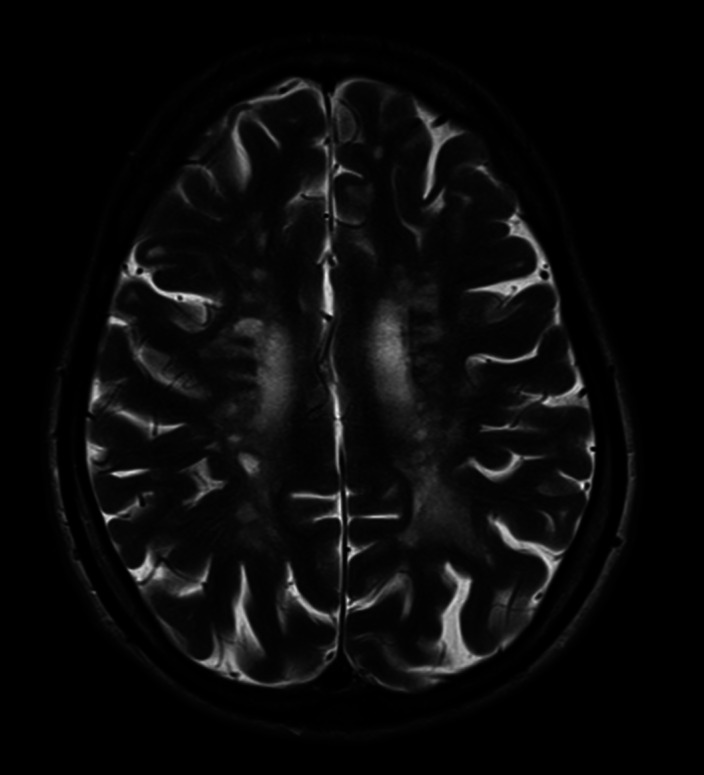

**Image 2:**

**Figure:**
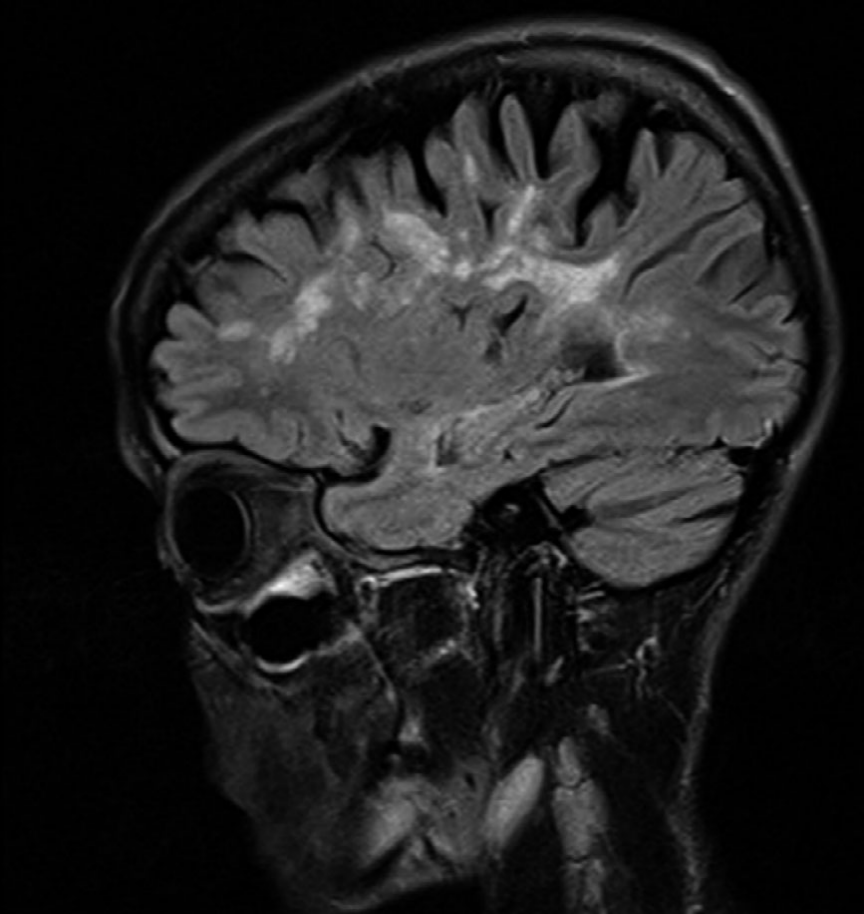

**Conclusions:**

The reformulation of the concept of cyclothymia would allow us to recognize in our patient a basic temperament of long evolution that would be the substrate on which different factors have subsequently influenced, such as antidepressant drugs or multiple sclerosis. In addition, it is necessary to know the association between BD and MS, in order to be able to offer an adequate treatment, contemplating some pharmacological options such as Lithium or some Atypical Antipsychotics, given the beneficial effect both for the affective disorder and for the neurological process.

**Disclosure of Interest:**

None Declared

